# Beyond Single Systems: How Multi-Agent AI Is Reshaping Ethics in Radiology

**DOI:** 10.3390/bioengineering12101100

**Published:** 2025-10-13

**Authors:** Sara Salehi, Yashbir Singh, Parnian Habibi, Bradley J. Erickson

**Affiliations:** 1 Radiology Informatics Lab, Department of Radiology, Mayo Clinic, Rochester, MN 55905, USA; salehi.sara@mayo.edu; 2 Department of Radiology, Mayo Clinic, Rochester, MN 55905, USA; singh.yashbir@mayo.edu (Y.S.); habibi.parnian@mayo.edu (P.H.)

**Keywords:** AI agents, explainable AI, radiology, transparency, clinical decision-making

## Abstract

Radiology is undergoing a paradigm shift from traditional single-function AI systems to sophisticated multi-agent networks capable of autonomous reasoning, coordinated decision-making, and adaptive workflow management. These agentic AI systems move beyond simple pattern recognition to encompass complex radiological workflows including image analysis, report generation, clinical communication, and care coordination. While multi-agent radiological AI promises enhanced diagnostic accuracy, improved workflow efficiency, and reduced physician burden, it simultaneously amplifies the long-standing “black box” problem. Traditional explainable AI methods, which are adequate for understanding isolated diagnostic predictions, fail when applied to multi-step reasoning processes involving multiple specialized agents coordinating across imaging interpretation, clinical correlation, and treatment planning. This paper examines how agentic AI systems in radiology create “compound opacity” layers of inscrutability from agent interactions and distributed decision-making processes. We analyze the autonomy–transparency paradox specific to radiological practice, where increasing AI capability directly conflicts with interpretability requirements essential for clinical trust and regulatory oversight. Through examination of emerging multi-agent radiological workflows, we propose frameworks for responsible implementation that preserve both diagnostic innovation and the fundamental principles of medical transparency and accountability.

## 1. Introduction

Radiology artificial intelligence (AI) has evolved from basic pattern recognition tools capable of identifying abnormalities to sophisticated agentic systems that can work through diagnostic problems using selected tools, execute multi-step reasoning processes, and coordinate with other specialized agents in integrated diagnostic workflows [1]. This transformation represents more than technological advancement. It is a fundamental shift in how AI systems take part in radiological practice, moving from passive diagnostic aids to active participants in clinical decision-making processes.

### Key Terminology: Before Proceeding with Our Analysis, We Define Two Central Concepts That Frame Our Discussion

Agentic AI Systems: We define agentic AI as artificial intelligence systems that exhibit autonomous goal-directed behavior, environmental perception, multi-step reasoning, tool utilization, and the capacity for coordinated action with other agents. Unlike traditional AI tools that perform specific pattern recognition tasks, agentic systems can independently plan diagnostic strategies, execute complex analytical workflows, and adapt their approaches based on feedback and contextual factors [2,3].

Compound Opacity: We introduce this term to describe the multiplicative inscrutability that emerges when multiple opaque AI agents interact within coordinated systems. Compound opacity differs from traditional “black box” opacity in that it encompasses not only individual agent reasoning processes but also inter-agent communication protocols, decision aggregation mechanisms, emergent system behaviors, and temporal dependencies that arise from agent coordination. This creates layers of complexity that exceed the sum of individual agent opacities [4].

Modern agentic AI systems in radiology show capabilities that extend far beyond traditional computer-aided detection (CAD) systems. These agents can analyze medical images using advanced image processing algorithms, integrate findings with clinical data through large language models (LLMs), develop diagnostic strategies, execute complex analyses step by step, and collaborate with other AI agents across the radiological workflow [1,2]. For instance, an agentic radiology system might independently identify suspicious findings on a CT scan, automatically correlate these with prior imaging studies, generate differential diagnoses, recommend appropriate follow-up protocols, and communicate findings to referring clinicians, all while adapting its approach based on patient-specific factors and real-time feedback.

However, this evolution toward autonomous, multi-agent systems in radiology has created unprecedented challenges for clinical transparency and accountability. As AI agents become more sophisticated in their reasoning and more autonomous in their decision-making, they simultaneously become more opaque and difficult to understand [2]. The problem compounds when multiple agents work together: radiological interpretation agents collaborating with clinical correlation agents, reporting agents working with communication systems, and workflow management agents coordinating follow-up procedures. Each decision point adds layers of complexity, making the entire diagnostic process increasingly inscrutable to human oversight [2,3].

The stakes of this opacity problem are particularly high in radiology, where diagnostic accuracy directly changes patient outcomes and where regulatory oversight demands clear accountability for medical decisions. Radiologists must be able to understand and confirm AI recommendations to support their role as the final arbiters of imaging interpretation. When AI systems use impenetrable black boxes or worse, as networks of interconnected black boxes, the fundamental trust relationship between radiologists and their diagnostic tools becomes compromised [5].

This paper addresses the critical challenges arising from the deployment of multi-agent AI systems in radiological practice. We examine how the shift from single-agent to multi-agent architectures amplifies existing transparency problems, analyze the unique ethical implications for radiological practice, and propose frameworks for responsible development that preserve both innovation benefits and essential principles of medical accountability.

## 2. The Evolution from Single-Agent to Multi-Agent Radiology AI

### 2.1. Traditional Radiological AI: The Single-Agent Paradigm

Early radiological AI systems operated as single-function tools designed to find specific pathological findings within narrowly defined parameters. These systems, exemplified by CAD tools for mammography screening or chest X-ray interpretation, provided binary classifications or confidence scores for diagnostic questions. While these single-agent systems had limitations in scope and flexibility, their decision-making processes were straightforward to characterize and confirm.

The interpretability challenge in single-agent radiological AI centered primarily on understanding feature attribution—finding which pixels or image regions contributed most strongly to diagnostic predictions. Techniques such as saliency mapping, gradient-based visualization, and attention mechanisms provided radiologists with visual explanations of AI decision-making, albeit with acknowledged limitations in clinical utility and reliability [6,7].

### 2.2. The Emergence of Agentic Radiological AI

The transition to agentic AI in radiology represents a qualitative shift from pattern recognition to autonomous reasoning and action. Agentic radiological systems demonstrate several key capabilities that distinguish them from traditional AI tools: environmental perception beyond single-image analysis, goal-directed behavior that adapts to clinical context, multi-step reasoning that integrates diverse information sources, tool utilization that extends beyond image processing, and collaborative coordination with other specialized agents [1,2].

Consider a contemporary agentic radiology system analyzing a complex abdominal CT scan. Rather than simply flagging potential abnormalities, the system might independently correlate findings across multiple imaging series, access relevant prior studies from the picture archiving and communication system (PACS), integrate laboratory values and clinical history, generate a prioritized differential diagnosis, recommend specific follow-up protocols, and coordinate with scheduling systems to arrange appropriate procedures—all while documenting its reasoning process and adapting its approach based on institutional protocols and patient-specific factors.

### 2.3. Multi-Agent Coordination in Radiological Workflows

The full potential of agentic AI in radiology appears through coordinated multi-agent systems where specialized agents collaborate across the imaging workflow. A comprehensive radiological multi-agent system might include: imaging analysis agents specialized for different modalities (CT, MRI, ultrasound, nuclear medicine), clinical correlation agents that integrate imaging findings with laboratory data and clinical history, reporting agents that generate structured radiology reports, communication agents that interface with referring clinicians and care teams, workflow management agents that coordinate scheduling and follow-up procedures, and quality assurance agents that monitor diagnostic accuracy and workflow efficiency [8].

These agents run within an interconnected ecosystem where diagnostic insights from imaging analysis agents inform clinical correlation agents, whose assessments guide reporting agents in generating clinical recommendations. Meanwhile, workflow management agents coordinate necessary follow-up procedures, and communication agents ensure prompt delivery of critical findings to relevant care teams.

### 2.4. Deep Learning Foundations of Multi-Agent Systems

Before examining multi-agent coordination challenges, it is essential to understand the deep learning foundations upon which these agents are built. Recent advances in medical image analysis have demonstrated remarkable capabilities in specialized diagnostic tasks, establishing the technical groundwork for more complex agentic systems. Modern deep learning architectures have achieved impressive performance across diverse pathological imaging tasks. Transformer-based approaches, such as Swin-Transformer networks utilizing focal loss mechanisms, have shown effectiveness in identifying pathological subtypes of lung adenocarcinoma despite high morphological similarity and class imbalance [9]. Similarly, deep neural networks have enabled automated detection of tuberculosis bacilli in sputum smear images, demonstrating the capability of deep learning systems to perform specialized microscopic analysis tasks. The challenge of interpretability in these single-task systems presages the compound opacity problem in multi-agent architectures. Feature disentanglement methods, exemplified by FDTs (Feature Disentangled Transformers), attempt to create more interpretable representations for tasks such as squamous cell carcinoma grading [10]. Advanced architectures incorporating attention mechanisms and causal reasoning—such as CGAM (Causality Graph Attention Mamba) networks for esophageal pathology grading, and DCA-DAFFNet combining deformable fusion attention with adaptive feature fusion for laryngeal tumor assessment—represent efforts to build interpretability into model architectures rather than applying it post hoc [11].

Hybrid approaches that integrate multiple learning paradigms further illustrate the evolution toward agent-like capabilities. Networks such as ViT-AMC, which combines vision transformers with adaptive model fusion and multi-objective optimization, and MamlFormer, which employs manifold adversarial multi-modal learning guided by prior experience, demonstrate increasingly sophisticated reasoning capabilities within individual models [12]. These systems begin to exhibit characteristics of agentic behavior: the ability to integrate multiple information sources, adapt to different diagnostic contexts, and employ learned strategies for complex classification tasks.

However, as these sophisticated individual models transition from isolated diagnostic tools to coordinated agents within multi-agent systems, their inherent interpretability challenges become amplified through inter-agent interactions. An individual deep learning model’s opacity is already substantial when processing high-dimensional medical images through multiple hidden layers compounds multiplicatively when multiple such models must coordinate their outputs, exchange intermediate representations, and collectively arrive at clinical recommendations. The attention mechanisms, feature disentanglement methods, and interpretability approaches developed for single models provide insufficient insight into the emergent behaviors that arise when these models interact as agents within complex diagnostic workflows. This transition from single sophisticated models to coordinated multi-agent systems represents not merely a quantitative increase in complexity but a qualitative shift in the nature of the interpretability challenge, which we explore in the following sections.

### 2.5. Emerging Clinical Implementations of Multi-Agent Radiology AI

While comprehensive multi-agent radiological systems remain largely in development, several emerging implementations demonstrate the trajectory toward coordinated AI workflows: Large Language Model Integration: Recent advances in applying large language models (LLMs) to radiology demonstrate agentic capabilities, including report generation, clinical correlation, and communication tasks [13]. These systems can analyze radiology reports, extract clinically relevant information, generate differential diagnoses, and communicate findings in natural language—capabilities that form foundational components of multi-agent architectures.

Workflow Orchestration Systems: Commercial platforms are beginning to deploy coordinated AI agents for workflow management, including triage systems that prioritize studies based on clinical urgency, quality assurance agents that monitor technical parameters, and communication agents that alert clinicians to critical findings. While these currently operate with significant human oversight, they demonstrate the feasibility of multi-agent coordination in clinical radiology environments [2,3].

Multi-Modal Integration Platforms: Several research implementations have demonstrated agents that coordinate across imaging modalities. For example, systems that integrate CT, PET, and clinical data for oncology applications employ specialized agents for each data type, with coordination mechanisms that synthesize findings into unified assessments. Early clinical pilots have shown both the potential benefits and the interpretability challenges we discuss throughout this review. These implementations, while not yet fully autonomous multi-agent systems, illustrate the practical trajectory and near-term challenges that motivate our analysis [1,8].

## 3. The Compound Opacity Problem in Multi-Agent Radiology

### 3.1. Beyond Traditional Black Box Challenges

Defining Compound Opacity

The transition from single-agent to multi-agent radiological AI has transformed the traditional “black box” problem into something fundamentally more complex and intractable. We define compound opacity as the multiplicative inscrutability that emerges when multiple opaque AI agents interact, creating layers of complexity that exceed traditional black-box problems in both scope and nature [1,4,8]. To clarify this distinction, Table 1 contrasts the characteristics of single-agent opacity with compound opacity in multi-agent systems:

The Nature of Compound Opacity

In single-agent radiological AI, explainability efforts focused on understanding how individual models processed imaging data to generate diagnostic predictions. Techniques like saliency mapping could highlight image regions most influential to AI decisions, providing radiologists with visual explanations of algorithmic reasoning [1,3,8]. While these explanations had significant limitations, including instability, lack of clinical correlation, and questionable fidelity to actual model reasoning, they at least offered a conceptual framework for understanding AI decision-making within a bounded system.

Multi-agent radiological systems shatter this framework. When multiple specialized agents collaborate to generate diagnostic recommendations, understanding system behavior requires understanding not only individual agent reasoning but also:

1. **Inter-agent communication protocols**: How agents exchange information, what representations they share, and how they interpret messages from other agents;
2. **Decision aggregation mechanisms**: How individual agent outputs combine to produce system-level recommendations;
3. **Emergent behaviors**: System-level patterns that arise from agent interactions but cannot be predicted from individual agent specifications;
4. **Temporal dependencies**: How agent decisions evolve based on information received from other agents over time;
5. **Context-dependent coordination**: How agent collaboration patterns adapt to different clinical scenarios.

A radiologist seeking to understand why a multi-agent system recommended a particular follow-up protocol must trace reasoning across multiple agents, each with its own decision logic and information processing approach. This tracing requires not only understanding what each agent concluded, but why agents reached those conclusions, how they communicated findings to other agents, and how the system aggregated potentially conflicting recommendations into a unified clinical plan.

Opacity compounds because each layer of agent interaction introduces additional inscrutability. If Agent A’s reasoning is 80% interpretable and Agent B’s reasoning is similarly 80% interpretable, their interaction does not yield 80% system interpretability [4,5]. Instead, understanding requires interpreting Agent A’s reasoning (80%), interpreting Agent B’s reasoning (80%), understanding how A’s output influenced B’s input (new complexity), understanding how B’s feedback modified A’s subsequent reasoning (additional complexity), and understanding emergent patterns that arise from their coordination (further complexity). The result is compound opacity multiplicative rather than additive complexity that renders traditional explainability methods inadequate.

### 3.2. The Failure of Current Explainability Methods

Traditional explainable AI methods, developed for single-decision outputs, prove inadequate for multi-agent radiological systems. Attention maps and attribution techniques can illuminate individual agent decisions but offer no insight into how these decisions aggregate into system-level recommendations. When an imaging analysis agent finds suspicious findings, a clinical correlation agent assesses their significance, and a reporting agent determines appropriate language and recommendations, the resulting diagnostic report reflects a complex negotiation process that current explainability methods cannot capture [14].

Moreover, the dynamic nature of multi-agent interactions creates temporal dependencies that static explanation methods cannot address. Agent decisions evolve based on information received from other agents, creating feedback loops and iterative refinement processes that unfold over time. Understanding why a multi-agent system recommended a particular diagnostic workup requires tracing these temporal interactions—a task that exceeds the capability of current interpretability frameworks [8].

### 3.3. Error Propagation and Cascading Failures

Multi-agent radiological systems introduce new categories of failure modes that are particularly concerning in clinical contexts. When agents operate in sequence—as when imaging analysis informs clinical correlation, which guides reporting decisions—errors can propagate and amplify throughout the diagnostic process. An initial misinterpretation by an imaging analysis agent might lead a clinical correlation agent to pursue incorrect diagnostic pathways, resulting in inappropriate recommendations that reflect compounded rather than isolated errors [8].

The mathematics of error propagation in multi-agent systems is sobering. If individual agents achieve 95% accuracy, a performance level that would be considered excellent in single-agent contexts, a five-agent sequential workflow achieves only 77% system-level accuracy (0.95^5). In radiological practice, where diagnostic accuracy directly impacts patient outcomes, such degradation in system reliability raises serious concerns about the safety and appropriateness of multi-agent approaches.

These technical challenges create broader implications for clinical practice.

### 3.4. Clinical Manifestations of Compound Opacity

To illustrate how compound opacity manifests in real-world radiological practice, we examine three representative clinical scenarios that demonstrate the practical implications of multi-agent system inscrutability [15].

Scenario 1: Lung Cancer Staging with Multi-Modal Integration

Consider a multi-agent system tasked with staging a newly diagnosed lung cancer:

**Agent Workflow:**

1. **Imaging Analysis Agent (CT)** analyzes chest CT and identifies:

- Primary tumor in right upper lobe (3.2 cm);

- Three ipsilateral mediastinal lymph nodes with suspicious morphology;

- Potential pleural nodule.

2. **Imaging Analysis Agent (PET)** processes PET/CT fusion data:

- Assigns SUV max values to identified structures;

- Flags additional bone lesion (L3 vertebra) showing FDG uptake;

- Notes limited uptake in one of the three lymph nodes.

3. **Clinical Correlation Agent** integrates laboratory and clinical data:

- Reviews tumor markers (elevated CEA);

- Incorporates pulmonary function tests;

- Accesses smoking history and comorbidities;

- Notes patient’s age and performance status.

4. **Staging Agent** synthesizes inputs to propose stage:

- Integrates anatomic and metabolic findings;

- Applies AJCC staging criteria;

- Generates preliminary stage: T2a N2 M1b (Stage IVA).

5. **Treatment Planning Agent** recommends management:

- Suggests systemic therapy rather than surgical resection;

- Proposes specific chemotherapy regimen based on staging;

- Recommends brain MRI for complete staging.

**Compound Opacity Manifestation:**

When the treating oncologist questions why surgical resection was not recommended, tracing the reasoning reveals multiple opacity layers:

- The CT agent’s confidence threshold for the pleural nodule is unclear (Was it definitively malignant or suspicious? What features drove that determination?).

- The PET agent’s interpretation of the bone lesion uptake involved proprietary SUV normalization algorithms.

- The clinical correlation agent weighted performance status heavily, but its weighting schema is opaque.

- The staging agent’s integration of conflicting signals (three lymph nodes on CT, but only two FDG-avid on PET) involved undocumented decision rules.

- The treatment planning agent’s recommendation reflected learned patterns from training data, but the specific cases that influenced this recommendation are unrecoverable.

A radiologist attempting to validate this recommendation cannot simply review a saliency map. They must: reconstruct the reasoning of five distinct agents, understand how uncertainty propagated through the system (Was the pleural nodule ambiguity communicated to downstream agents?), identify which factors were most influential to the final recommendation (Was it the bone lesion? The staging? The performance status?), and assess whether alternative interpretations were considered and appropriately weighted. The compound opacity is particularly problematic because each agent’s intermediate conclusion seems individually reasonable, yet the radiologist cannot determine whether the system-level recommendation optimally integrates all available information or whether errors of interpretation or communication led to a suboptimal plan.

Scenario 2: Emergency CT Triage for Stroke [16]

A multi-agent system managing emergency CT interpretation for potential stroke patients illustrates how compound opacity affects time-critical decisions:

**Agent Workflow:**

1. **Triage Agent** evaluates incoming study requisitions:

- Assigns priority based on clinical indication;

- Places “suspected stroke” studies in expedited queue;

- Coordinates with scanner availability and technologist workflow.

2. **Image Quality Agent** assesses acquired images:

- Evaluates motion artifact;

- Checks coverage adequacy;

- Flags technical issues requiring repeat acquisition.

3. **Analysis Agent (Non-contrast CT)** performs automated interpretation:

- Measures ASPECTS score;

- Identifies early ischemic changes;

- Quantifies hyperdense vessel signs.

4. **Analysis Agent (CT Angiography)** evaluates vascular imaging:

- Identifies large vessel occlusion (M1 segment);

- Assesses collateral circulation;

- Generates automated vessel maps.

5. **Analysis Agent (CT Perfusion)** processes perfusion data:

- Calculates ischemic core volume;

- Estimates penumbral tissue at risk;

- Computes mismatch ratios.

6. **Integration Agent** synthesizes findings:

- Combines structural, vascular, and perfusion data;

- Applies learned decision rules for thrombectomy candidacy;

- Generates preliminary recommendation.

7. **Communication Agent** alerts clinical team:

- Determines urgency level;

- Selects appropriate notification channels;

- Formats findings for rapid review.

**Compound Opacity Manifestation:**

When a patient experiences a poor outcome despite thrombectomy, the neurologist questions whether the system’s recommendation was appropriate. Investigation reveals:

- The triage agent delayed the study by 8 min to accommodate a “higher priority” trauma case, but its priority algorithm is proprietary.

- The image quality agent approved images with moderate motion artifact that may have affected ASPECTS scoring.

- The non-contrast analysis agent’s ASPECTS score (7) differed from the perfusion-derived core volume, suggesting inconsistent assessments of infarct extent.

- The CTA agent identified good collaterals, but its collateral scoring method was trained on a different population than the local patient demographic.

- The perfusion agent used vendor-specific post-processing with undisclosed temporal sampling parameters.

- The integration agent weighted the perfusion mismatch heavily, potentially overriding conflicting signals from the ASPECTS score.

- The communication agent sent a “code stroke” alert, but its urgency classification criteria are undocumented.

The radiologist reviewing the case cannot determine: whether the 8 min triage delay reflected appropriate prioritization, whether the image quality issues meaningfully affected downstream interpretations, why the ASPECTS and perfusion-derived core estimates diverged, whether the collateral assessment was reliable for this patient population, whether the perfusion analysis used appropriate parameters for this scanner and contrast protocol, how the integration agent resolved conflicting inputs, or whether an alternative synthesis of the available data would have led to a different recommendation.

The compound opacity is especially concerning in emergency scenarios because temporal pressures preclude detailed investigation of system reasoning, yet the stakes of errors are highest. Radiologists must decide whether to trust opaque recommendations in time-critical situations where traditional validation approaches are infeasible.

Scenario 3: Multidisciplinary Tumor Board Preparation [17]

Multi-agent systems that prepare comprehensive case presentations for tumor boards demonstrate compound opacity in clinical communication contexts:

**Agent Workflow:**

1. **Case Identification Agent** scans radiology information system:

- Identifies patients with new cancer diagnoses;

- Flags cases requiring multidisciplinary discussion;

- Schedules tumor board presentations.

2. **Imaging Compilation Agent** aggregates relevant studies:

- Retrieves current diagnostic imaging;

- Identifies relevant prior studies;

- Organizes chronologically for comparison.

3. **Analysis Agents** (multiple, modality-specific) interpret studies:

- Generate structured findings for each imaging examination;

- Measure lesions and track changes over time;

- Apply disease-specific protocols (e.g., RECIST criteria).

4. **Clinical Context Agent** reviews electronic health record:

- Extracts relevant history, laboratory values, and pathology results;

- Identifies prior treatments and responses;

- Summarizes performance status and comorbidities.

5. **Literature Agent** searches database:

- Identifies relevant clinical trials;

- Retrieves treatment guidelines;

- Flags recent publications on similar cases.

6. **Synthesis Agent** generates comprehensive presentation:

- Integrates imaging findings with clinical context;

- Proposes differential diagnoses or staging assessments;

- Suggests treatment options based on guidelines and literature;

- Formats information for tumor board discussion.

**Compound Opacity Manifestation:**

When tumor board participants question the agent-generated presentation, several opacity issues emerge:

- The case identification agent flagged this patient for discussion, but other potentially appropriate cases were not included—the selection criteria are unclear.

- The imaging compilation agent retrieved 12 prior studies but not a 3-year-old outside MRI that the referring oncologist mentions—why was it missed?

- The analysis agents measured response using RECIST 1.1, but for this immune therapy case, iRECIST might have been more appropriate—why was this choice made?

- The clinical context agent summarized “good performance status” despite documented recent weight loss—how were conflicting indicators weighted?

- The literature agent identified three relevant trials but missed a recently published study the medical oncologist knows about—what search strategy was used?

- The synthesis agent proposed a treatment approach that seems reasonable but differs from what a specialist would recommend—what factors drove this suggestion?

The radiologist preparing for tumor board cannot adequately validate the presentation because the case selection process is opaque (Are we missing important cases?), the imaging compilation logic is unclear (What studies were excluded and why?), the measurement and assessment approaches may not align with specialty-specific best practices, the clinical context summarization involves subjective weighting, the literature search strategy and recency are unknown, and the synthesis reflects learned patterns that may not capture cutting-edge approaches.

Compound opacity is particularly problematic in multidisciplinary settings because it affects not just diagnostic accuracy but clinical communication and collaborative decision-making. When specialists from different disciplines question AI-generated presentations, radiologists cannot provide the detailed explanations needed to build confidence in the system’s comprehensive assessments.

Implications for Clinical Practice

These scenarios illustrate several common patterns in how compound opacity manifests:

1. **Cascading uncertainty**: Initial ambiguities or low-confidence assessments propagate through agent chains, but confidence levels may not be appropriately communicated or preserved.

2. **Inconsistency reconciliation**: When different agents provide conflicting information, the mechanisms for resolving discrepancies are opaque.

3. **Context sensitivity**: Agent decisions depend on training data, institutional protocols, and population characteristics in ways that are not transparent to end users.

4. **Temporal complexity**: Sequential and iterative agent interactions create path dependencies that are difficult to reconstruct retrospectively.

5. **Communication fidelity**: Information passed between agents may lose important nuance or context, affecting downstream reasoning.

For radiologists, compound opacity in these scenarios creates practical challenges: inability to validate recommendations with available time and cognitive resources, difficulty communicating the basis for AI-assisted decisions to clinical colleagues, limited capacity to identify and correct errors before they affect patient care, and reduced ability to learn from cases to improve future practice.

These real-world implications underscore why compound opacity represents more than a theoretical concern. It directly affects the quality of patient care and the professional practice of radiology in ways that demand urgent attention from developers, practitioners, and regulatory bodies.

## 4. Autonomy–Transparency Tensions in Radiological Practice

### 4.1. The Clinical Need for Understanding

Radiology practice fundamentally depends on diagnostic confidence and clinical judgment that require deep understanding of the reasoning processes underlying diagnostic recommendations. Radiologists must be able to assess the reliability of AI-generated findings, integrate them with clinical context, and communicate diagnostic uncertainty appropriately to referring physicians and patients [18]. This clinical requirement for understanding creates a direct tension with the increasing opacity of multi-agent AI systems.

The problem extends beyond individual diagnostic decisions to encompass broader questions of clinical competence and professional responsibility. Radiologists who cannot understand the reasoning behind AI recommendations face difficult choices between blind acceptance of algorithmic guidance and complete rejection of potentially valuable insights. Neither option serves patient interests effectively, and both undermine the radiologist’s role as the ultimate diagnostic authority.

### 4.2. Regulatory and Legal Implications

The opacity of multi-agent radiological AI systems creates significant challenges for regulatory oversight and legal accountability. Current medical device regulations assume that system behavior can be characterized, validated, and audited through established testing protocols. Multi-agent systems with emergent behaviors and adaptive capabilities challenge these assumptions, creating regulatory gaps that could impede clinical deployment or, worse, allow unsafe systems to enter clinical practice without adequate oversight [19].

Legal accountability becomes particularly complex when diagnostic errors occur in multi-agent systems. Determining whether errors stem from individual agent failures, inter-agent communication breakdowns, or emergent system behaviors requires forensic capabilities that currently do not exist. This attribution problem has implications not only for medical liability but also for quality improvement efforts that depend on understanding failure modes to prevent recurrence.

#### 4.2.1. Jurisdictional Differences in Regulatory Approaches

Regulatory frameworks for medical AI vary significantly across jurisdictions, and these differences become particularly salient for multi-agent systems whose behavior may be difficult to characterize through traditional validation methods.

In the United States, the Food and Drug Administration (FDA) has primarily regulated medical AI through the 510(k) premarket notification pathway, which relies on demonstrating substantial equivalence to predicate devices. However, this framework was designed for static medical devices with predictable behavior and struggles to accommodate adaptive multi-agent systems whose reasoning processes and inter-agent coordination patterns may evolve over time. The FDA’s proposed approach for AI/ML-based Software as a Medical Device (SaMD) acknowledges these challenges and proposes predetermined change control plans, but fundamental questions remain about how to validate systems whose behavior emerges from agent interactions rather than predefined algorithms [20].

The European Medicines Agency (EMA) and European regulatory framework place greater emphasis on clinical evaluation and post-market surveillance. Under the Medical Device Regulation (MDR) and In Vitro Diagnostic Regulation (IVDR), AI systems must demonstrate clinical benefit through rigorous evaluation processes. For multi-agent systems, this clinical evaluation requirement is particularly challenging because emergent behaviors may not be apparent during initial validation, and inter-agent coordination patterns may vary across clinical contexts in ways that are difficult to predict or systematically evaluate before deployment [21].

The European Union’s AI Act introduces an additional regulatory layer, classifying medical AI systems as “high-risk” applications subject to stringent transparency and accountability requirements. The AI Act’s emphasis on explainability, human oversight, and technical documentation aligns closely with the concerns we identify regarding compound opacity in multi-agent systems. However, the Act’s requirements—including the mandate that high-risk AI systems be “sufficiently transparent to enable users to interpret the system’s output and use it appropriately”—may prove difficult to satisfy for complex multi-agent architectures. How regulators will assess compliance with transparency requirements for systems exhibiting compound opacity remains an open question [22].

Importantly, these regulatory frameworks were largely developed before the emergence of sophisticated multi-agent medical AI and do not explicitly address key challenges such as: validating emergent system behaviors that arise from agent interactions rather than programmed algorithms, assessing the safety and reliability of systems with distributed decision-making authority, establishing accountability when errors result from inter-agent communication failures rather than individual model mistakes, monitoring post-deployment system behavior when agents adapt their coordination patterns, and ensuring transparency when explanations must span multiple agents and decision layers.

The regulatory uncertainty surrounding multi-agent medical AI creates both risks and opportunities. Systems might enter clinical practice without adequate validation if regulators lack frameworks to assess them appropriately, or conversely, regulatory caution might impede valuable innovations that could improve patient care. Developing regulatory approaches adequate to multi-agent systems requires close collaboration among regulatory agencies, AI developers, medical practitioners, and ethicists to balance innovation with patient safety [8,21,22].

#### 4.2.2. Pathways for Regulatory Adaptation

Adapting existing regulatory frameworks to multi-agent medical AI systems requires addressing several unresolved challenges:

Pre-Market Validation Challenges: Traditional validation approaches assess AI systems using fixed test datasets with ground truth labels. Multi-agent systems present challenges for this paradigm: How should regulators validate emergent behaviors that arise from agent interactions rather than programmed algorithms? Should each agent be validated independently, or must the entire multi-agent system undergo holistic validation? When agents adapt their coordination patterns based on clinical experience, what triggers re-validation requirements? [20,21]

Post-Market Surveillance Requirements: Effective oversight of deployed multi-agent systems requires continuous monitoring capabilities that exceed current post-market surveillance frameworks. Regulatory agencies must develop standards for (1) logging and audit trail requirements that capture inter-agent communications and decision processes, (2) performance metrics that assess system-level behaviors rather than individual agent accuracy, and (3) mandatory reporting triggers when multi-agent coordination patterns deviate from validated behaviors.

Accountability Frameworks: Legal liability attribution in multi-agent contexts requires new frameworks. Current medical device liability focuses on manufacturer responsibility for device failures or physician responsibility for usage errors. When errors emerge from complex agent interactions rather than individual component failures, determining appropriate accountability becomes challenging. Should liability rest with the developers of individual agents, the integrator who coordinated them into a multi-agent system, the healthcare institution that deployed the system, or the radiologist who relied on system recommendations?

International Harmonization Needs: The global nature of AI development and deployment makes international regulatory harmonization increasingly important. However, jurisdictional differences in transparency requirements, validation standards, and accountability frameworks could create barriers to multi-agent system deployment. For instance, systems designed to meet EU AI Act transparency requirements may face different validation challenges under FDA frameworks [21,22]. Developing harmonized standards while respecting legitimate jurisdictional differences represents a critical policy challenge. Proposed Regulatory Approaches Several promising approaches could address these challenges [21,22]:

1. Adaptive Regulatory Frameworks: Rather than static pre-market approval, regulators could implement predetermined change control plans that specify acceptable ranges of agent adaptation and coordination patterns, with continuous performance monitoring and mandatory reporting when systems approach specified boundaries.

2. Sandbox Environments: Regulatory sandboxes could allow controlled clinical deployment of multi-agent systems under close oversight, enabling empirical evidence gathering about safety and effectiveness while protecting patient welfare through enhanced monitoring and rapid intervention capabilities.

3. Certification of Development Processes: Rather than attempting to validate all possible multi-agent behaviors, regulators could certify development processes, testing methodologies, and post-deployment monitoring capabilities that provide reasonable assurance of safety even when specific system behaviors cannot be fully predicted. These unresolved challenges highlight the urgency of regulatory innovation to keep pace with technological advancement while ensuring patient safety.

### 4.3. Trust and Professional Identity

The relationship between radiologists and AI systems fundamentally depends on trust—specifically, calibrated trust that appropriately balances reliance on AI capabilities with recognition of system limitations [23,24]. Multi-agent opacity undermines this trust relationship by making it impossible for radiologists to assess when AI recommendations are likely to be dependable and when they should be viewed with skepticism.

This trust deficit has implications beyond individual diagnostic decisions. It affects the professional identity of radiologists and their perceived value in clinical care delivery. If AI systems become so sophisticated that they can manage complex diagnostic tasks autonomously, but so opaque that radiologists cannot understand or validate their reasoning, the radiologist’s role becomes unclear. Are they merely rubber-stamping algorithmic decisions, or do they retain meaningful diagnostic authority?

Table 2 summarizes the multi-agent challenges discussed in Section 3 and Section 4, along with their clinical impacts and proposed solutions.

Having established the fundamental tensions between autonomy and transparency in radiological practice (Section 4.1, Section 4.2 and Section 4.3), we now turn to frameworks and technical approaches for addressing these challenges. Section 5 proposes ethical principles for responsible development, while Section 6 examines specific technical solutions for improving multi-agent transparency.

## 5. Ethical Frameworks for Multi-Agent Radiology AI

The challenges identified in Section 3 and Section 4—compound opacity, error propagation, accountability gaps, and trust deficits—require systematic responses at multiple levels. We organize our proposed solutions into three categories: ethical frameworks (Section 5), technical approaches (Section 6), and implementation strategies (Section 7).

### 5.1. Preserving Human Agency in Diagnostic Decision-Making

The deployment of autonomous multi-agent systems in radiology must preserve meaningful human agency in diagnostic decision-making while leveraging the capabilities of AI to improve care quality and efficiency. This requires moving beyond simple human-in-the-loop models toward more sophisticated frameworks that support genuine human–AI collaboration in diagnostic reasoning.

One promising approach involves role separation, where AI agents manage well-defined technical tasks while radiologists maintain responsibility for clinical interpretation, diagnostic synthesis, and communication with referring physicians [25]. In this model, imaging analysis agents might autonomously identify and characterize imaging findings, but radiologists retain authority over diagnostic conclusions and clinical recommendations. This preserves human agency while enabling AI systems to contribute their analytical capabilities to the diagnostic process.

### 5.2. Transparency Requirements and Design Principles

Multi-agent radiological AI systems must be designed with transparency as a fundamental requirement rather than an afterthought. This involves implementing hierarchical explanation mechanisms that operate at multiple levels: individual agent reasoning, inter-agent communication, and system-level decision aggregation. Each level of explanation should be tailored to the needs of different stakeholders: detailed technical explanations for system developers, clinical explanations for radiologists, and summary explanations for referring physicians and patients.

Natural language communication between agents, as proposed in multi-agent healthcare architectures, offers one promising approach to enhancing transparency [26]. When agents communicate using human-understandable language, their interactions become more interpretable to human oversight. Additionally, such communication creates audit trails that can be reviewed and analyzed when questions arise about system decision-making.

### 5.3. Accountability Mechanisms and Error Attribution

Multi-agent radiological systems require sophisticated accountability mechanisms that can attribute responsibility appropriately when errors occur. This involves not only technical capabilities for tracing decision processes but also clear frameworks for assigning responsibility among human and artificial agents. When multiple AI agents contribute to a diagnostic recommendation, and that recommendation proves incorrect, determining appropriate accountability requires understanding both the technical failure modes and the human oversight processes that should have detected and corrected the error [8].

Comprehensive logging and audit trail systems are essential for supporting accountability in multi-agent environments. These systems must capture not only final decisions but also the reasoning processes, information sources, and interaction patterns that led to those decisions. Such detailed documentation enables post hoc analysis of errors and supports continuous improvement of both technical systems and human oversight processes.

Implementing these ethical principles demands specific technical solutions.

## 6. Technical Approaches to Multi-Agent Transparency

### 6.1. Hierarchical Explanation Architectures

Addressing the compound opacity of multi-agent radiological systems requires explanation architectures that operate at multiple levels of abstraction. Individual agent explanations might focus on specific technical decisions—why an imaging analysis agent flagged findings or how a clinical correlation agent weighted different diagnostic possibilities. Inter-agent explanations should illuminate communication patterns and information flow between agents. System-level explanations must synthesize these components into coherent narratives that help radiologists understand overall system reasoning [8].

Such hierarchical approaches demand careful attention to cognitive load and information presentation. Radiologists cannot be expected to understand detailed technical explanations of every agent’s decision in a complex multi-agent workflow. Instead, explanation systems must provide appropriate levels of detail for different context summary explanations for routine cases, detailed explanations when system confidence is low, and comprehensive explanations when errors occur or system recommendations are questioned.

### 6.2. Adversarial Agents and Internal Validation

One promising approach to improving multi-agent system reliability and transparency involves incorporating adversarial agents that explicitly challenge primary diagnostic recommendations. Just as radiological practice benefits from second opinions and multidisciplinary consultation, multi-agent systems could include specialized agents designed to identify potential errors, question diagnostic assumptions, and propose alternative interpretations.

These adversarial agents would operate using different algorithms, training data, or analytical approaches than primary diagnostic agents, providing a diversity of perspectives that could identify errors before they propagate through the system. Their challenges and alternative recommendations would be documented as part of the explanation process, providing radiologists with insight into areas of diagnostic uncertainty or disagreement within the multi-agent system.

### 6.3. Mechanistic Interpretability for Agent Understanding

Advances in mechanistic interpretability—techniques for understanding the internal reasoning processes of individual AI agents—offer potential pathways for improving multi-agent transparency [27]. By making individual agent reasoning more interpretable, these approaches could provide the foundation for understanding multi-agent interactions and system-level behaviors.

However, mechanistic interpretability faces significant challenges when applied to the large language models and complex neural networks that power modern agentic AI systems. Current techniques collaborate best with simple models and well-defined tasks. Extending these approaches to the complex, multi-modal reasoning required in radiological diagnosis represents a significant technical challenge that will require sustained research effort.

Translating these technical approaches into practice requires careful implementation strategies.

## 7. Implementation Pathways and Future Directions

### 7.1. Staged Deployment and Risk Management

The path toward multi-agent radiological AI should proceed through carefully designed stages that prioritize learning, safety, and trust-building. Initial implementations should focus on low-risk applications where multi-agent coordination can demonstrate value without compromising patient safety. Examples might include workflow optimization, routine screening protocols, or administrative task coordination where errors have limited clinical impact.

As experience accumulates and technical solutions mature, systems can gradually take on more complex and consequential diagnostic tasks. This staged approach allows for iterative improvement of both technical systems and human oversight processes while building confidence in multi-agent approaches among radiologists and healthcare institutions.

### 7.2. Education and Training Requirements

The successful integration of multi-agent AI into radiological practice requires comprehensive education and training programs that prepare radiologists to work effectively with these sophisticated systems. Traditional radiology training focuses on image interpretation and clinical correlation but provides limited preparation for understanding and validating AI-generated recommendations, particularly from complex multi-agent systems.

Training programs must address both technical and clinical aspects of multi-agent AI systems. Radiologists need sufficient technical understanding to assess system capabilities and limitations, identify potential errors, and maintain appropriate skepticism about AI recommendations. They also need clinical skills for integrating AI insights into diagnostic reasoning and communicating AI-assisted findings to referring physicians and patients.

### 7.3. Research Priorities and Knowledge Gaps

Several critical research areas require attention to support responsible multi-agent AI development in radiology. First, new evaluation methodologies are needed that assess multi-agent system performance beyond traditional accuracy metrics. These evaluations should include measures of transparency, reliability, error propagation, and impact on radiologist decision-making and workflow efficiency.

Second, empirical studies of human–AI collaboration in multi-agent contexts are essential for understanding how radiologists can effectively interact with and oversee complex AI systems. Such studies should examine not only diagnostic accuracy but also cognitive workload, trust calibration, and professional satisfaction among radiologists collaborating with multi-agent systems.

Third, the development of radiology-specific multi-agent interpretability methods represents a crucial technical challenge. Current explainable AI research focuses primarily on single models and simplified tasks. Extending these approaches to multi-agent systems managing complex radiological workflows requires new theoretical frameworks and methodological innovations tailored to the specific requirements of medical imaging and diagnostic reasoning. These implementation challenges raise fundamental questions about the future of radiological practice.

## 8. Discussion

### 8.1. Balancing Innovation and Responsibility

The emergence of multi-agent AI in radiology presents a classic dilemma between technological capability and human understanding. These systems offer genuine potential to improve diagnostic accuracy, reduce interpretive errors, and enhance workflow efficiency. However, their opacity and complexity challenge fundamental assumptions about human oversight and professional responsibility in medical practice.

The solution requires neither blind acceptance of technological capabilities nor reflexive resistance to innovation. Instead, it demands thoughtful approaches that preserve the benefits of human expertise while leveraging AI capabilities in clinically appropriate ways. This balance can only be achieved through sustained collaboration between technologists, radiologists, ethicists, and regulatory experts working together to develop systems that serve both technological and clinical objectives.

Healthcare professionals carry ethical obligations to maintain competence and take responsibility for clinical decisions. Multi-agent AI systems challenge these fundamental obligations by creating scenarios where radiologists may find themselves relying on recommendations they cannot fully evaluate or understand. Recognizing this tension, recent work has proposed role separation approaches that utilize the unique strengths of both AI and radiologists through complementary but distinct roles within diagnostic workflows, avoiding automation-related issues by dividing processes into distinct parts suited to each system’s capabilities [25]. While we acknowledge the validity of these concerns about human agency and accountability, our analysis suggests that emerging agentic AI systems may offer pathways toward enhanced human–machine collaboration through more dynamic and adaptive workflows. The critical factor lies in building transparency mechanisms and establishing continuous feedback loops—approaches that do not eliminate the risks of compound opacity but provide better frameworks for identifying and addressing them as they emerge. The path forward may require not choosing between separation and integration, but rather thoughtfully designing systems that harness the strengths of both human expertise and artificial intelligence while actively safeguarding against their respective limitations.

### 8.2. The Future of Radiological Practice

Multi-agent AI systems will transform radiological practice in fundamental ways, shifting radiologists toward more supervisory and integrative roles while AI agents manage routine technical tasks. However, this transformation should enhance rather than diminish the radiologist’s clinical value. By automating routine processes and providing sophisticated analytical capabilities, multi-agent systems could free radiologists to focus on complex diagnostic challenges, patient interaction, and clinical consultation that require human judgment and empathy.

The key to realizing this vision lies in designing multi-agent systems that complement rather than replace human expertise. This requires not only technical sophistication but also deep understanding of radiological workflows, clinical requirements, and professional values that define effective medical practice.

### 8.3. Broader Implications for Medical AI

The challenges identified in multi-agent radiological AI have implications beyond radiology for the broader field of medical AI. As AI systems become more sophisticated and autonomous across medical specialties, questions of transparency, accountability, and human oversight will become increasingly pressing. The frameworks and solutions developed for radiological applications could inform approaches in other medical domains facing similar challenges.

Moreover, the compound opacity problem identified in multi-agent systems represents a fundamental challenge for AI safety and alignment in high-stakes domains. The techniques and principles developed to address these challenges in radiology could contribute to broader efforts to ensure AI systems remain beneficial and aligned with human values as they become more capable and autonomous.

### 8.4. Limitations of This Review

This review has several important limitations that readers should consider when interpreting our analysis and recommendations.

**Primarily Conceptual Analysis**: Our examination of multi-agent AI systems in radiology is necessarily conceptual and theoretical, given the limited number of multi-agent radiological AI systems currently deployed in clinical practice. While we draw on principles from AI research, medical ethics, and radiological practice, empirical validation of many claims awaits broader clinical implementation of these systems. The scenarios presented illustrate potential challenges but are not based on systematic study of actual multi-agent system failures or successes in real-world settings.

**Limited Empirical Evidence Base**: The literature on multi-agent AI in radiology specifically remains sparse. While robust evidence exists for single-agent AI systems in imaging interpretation, empirical studies examining the compound opacity problem, error propagation patterns, trust calibration dynamics, and workflow integration challenges in multi-agent contexts are scarce. Our analysis extrapolates from related domains and theoretical considerations, but definitive evidence regarding the magnitude and nature of these challenges in radiological practice requires prospective study.

**Rapidly Evolving Field**: The landscape of AI technology and radiological applications is changing rapidly. Technical solutions to transparency and interpretability challenges continue to advance, and new multi-agent architectures emerge frequently. Findings and recommendations that seem prescient today may become outdated as technology evolves. Similarly, regulatory frameworks and professional guidelines for AI in radiology remain in active development, and future policy directions may address concerns we identify in ways we cannot fully anticipate.

**Radiology-Specific Focus**: While we believe many insights generalize to other medical specialties and high-stakes decision-making domains, our analysis focuses specifically on radiological practice. The unique characteristics of radiology—including image-based diagnosis, often limited patient interaction, integration with multiple clinical specialties, and heavy reliance on technology—may limit the applicability of our frameworks to other medical contexts. Domains with different practice patterns, accountability structures, or technical requirements may face distinct challenges requiring adapted approaches.

**Technical Depth Constraints**: Our review addresses both technical and ethical dimensions of multi-agent radiological AI but cannot provide exhaustive technical detail on all relevant topics. Readers seeking comprehensive understanding of specific technical approaches—such as mechanistic interpretability methods, adversarial training techniques, or federated learning architectures—should consult specialized literature in those domains. Similarly, legal and regulatory analysis is necessarily high-level; detailed policy guidance requires domain-specific expertise beyond the scope of this review.

**Proposed Solutions Require Validation**: The frameworks and technical approaches we propose for addressing compound opacity and preserving transparency in multi-agent systems represent informed recommendations based on current understanding, but they require empirical validation before being considered established best practices. Implementation challenges, unintended consequences, and practical limitations may emerge as these approaches are tested in clinical settings. We present these proposals as starting points for discussion and research rather than validated solutions.

**Assumption of Agent Coordination**: Our analysis assumes multi-agent systems in radiology will involve genuine agent coordination with sophisticated inter-agent communication and collaborative decision-making. If future implementations take simpler forms—such as parallel independent agents whose outputs are aggregated through straightforward rules—some challenges we identify may prove less severe. Conversely, if agent autonomy and interaction complexity exceed current projections, challenges may be more substantial than we describe.

**Limited Stakeholder Perspectives**: While we consider perspectives of radiologists, patients, referring clinicians, and developers, our analysis primarily reflects the radiologist’s viewpoint. More comprehensive examination would benefit from systematic input from patients regarding their preferences for AI involvement in diagnosis, referring physicians regarding their needs for understanding AI-assisted interpretations, hospital administrators regarding practical implementation considerations, and AI developers regarding technical feasibility constraints.

Despite these limitations, we believe this review provides a valuable framework for understanding the ethical and practical challenges posed by multi-agent AI in radiology and offers constructive pathways toward responsible development and deployment. The limitations identified here also highlight important directions for future empirical research and interdisciplinary collaboration needed to fully address the complex questions raised by increasingly sophisticated AI systems in medical practice.

## 9. Conclusions

The evolution of radiology AI from single-function tools to sophisticated multi-agent systems represents both a tremendous opportunity and significant challenge. While these systems promise improvements in diagnostic accuracy, workflow efficiency, and clinical capability, they also introduce unprecedented levels of complexity and opacity that challenge traditional frameworks for medical accountability and professional responsibility.

The compound opacity problem—where traditional explainability methods fail to illuminate the reasoning processes of interconnected AI agents—demands novel approaches to transparency, accountability, and human oversight in radiological practice. Addressing these challenges requires coordinated efforts across technical development, regulatory frameworks, professional education, and ethical guidelines.

The stakes of getting this transition right are significant. Multi-agent AI has the potential to transform radiological practice in ways that improve patient outcomes while enhancing rather than diminishing the professional role of radiologists. However, realizing this potential requires careful attention to the unique challenges posed by autonomous, coordinated AI systems used in clinical environments.

The future of radiology will likely be defined by our collective ability to develop and deploy multi-agent AI systems that preserve the essential human elements of medical practice, clinical judgment, professional responsibility, and patient-centered care, while leveraging the capabilities of artificial intelligence to improve diagnostic accuracy and efficiency. This requires not just technological innovation but also wisdom in navigating the complex ethical and practical challenges that arise when sophisticated AI systems become active participants in medical decision-making.

Success in this endeavor will require ongoing collaboration among radiologists, AI researchers, ethicists, and policymakers, working together to ensure that the evolution of radiological AI serves both technological advancement and the fundamental goals of medical practice: improving patient outcomes while maintaining the trust and human connections that define effective healthcare delivery.

## Figures and Tables

**Table 1:** Single-Agent Opacity vs. Compound Opacity in Multi-Agent Systems.

Dimension	Single-Agent Opacity	Compound Opacity (Multi-Agent)
**Primary Source**	Internal model complexity (neural network weights, activation patterns)	Agent interactions + individual model opacity + emergent system behaviors
**Explainability Target**	One decision pathway with identifiable input-output mapping	Multiple interacting decision pathways with distributed reasoning
**Transparency Methods**	Saliency maps, attention visualization, feature attribution	Requires hierarchical explanations across agent communications and decision aggregation
**Error Attribution**	Traceable to specific model components or training data	Distributed across multiple agents with unclear attribution
**Temporal Complexity**	Static decision point amenable to snapshot analysis	Dynamic iterative exchanges with temporal dependencies
**Validation Approach**	Test set evaluation with ground truth comparison	System-level behavior assessment requiring process monitoring
**Human Oversight**	Review individual model outputs	Monitor agent interactions and system-level emergent behaviors

**Table 2:** Multi-Agent Challenges and Proposed Solutions.

Challenge	Description	Clinical Impact	Proposed Solution	Implementation Section
**Compound Opacity**	Multiplicative inscrutability arising from inter-agent interactions, distributed reasoning, and emergent system behaviors (Section 3.1)	Radiologists cannot validate AI recommendations; uncertainty about reliability of multi-step diagnostic processes	Hierarchical explanation architectures providing agent-level, interaction-level, and system-level transparency	Section 6.1
**Explainability Method Failure**	Traditional attribution techniques (saliency maps, attention visualization) inadequate for multi-agent coordination (Section 3.2)	Inability to understand how individual agent decisions aggregate into system recommendations	Natural language agent communication; audit trails documenting reasoning processes	Section 5.2 and Section 6.1
**Error Propagation**	Cascading failures as errors amplify through sequential agent workflows; system accuracy degrades multiplicatively (Section 3.3)	Compounded diagnostic errors with unclear origin; reduced reliability despite high individual agent accuracy	Adversarial agents providing internal validation; diversity of analytical approaches	Section 6.2
**Attribution Difficulty**	Unclear responsibility when errors emerge from agent interactions rather than individual component failures (Section 3.3)	Legal liability ambiguity; challenges in quality improvement and root cause analysis	Comprehensive logging systems; accountability frameworks assigning responsibility across human and artificial agents	Section 5.3
**Trust Calibration Deficit**	Radiologists cannot assess when to rely on versus question AI recommendations (Section 4.3)	Over-reliance on flawed recommendations or under-utilization of valuable insights; professional identity concerns	Staged deployment in low-risk contexts; empirical validation studies; transparency mechanisms building calibrated trust	Section 7.1 and Section 7.2
**Regulatory Gaps**	Existing frameworks inadequate for validating emergent behaviors and adaptive multi-agent coordination (Section 4.2)	Unsafe systems potentially entering practice; valuable innovations impeded by regulatory uncertainty	Adaptive regulatory frameworks; sandbox environments; certification of development processes rather than all behaviors	Section 4.2
**Clinical Context Insensitivity**	Agent decisions reflect training data and protocols that may not match local patient populations or institutional practices (Section 3.4)	Recommendations inappropriate for specific clinical contexts; reduced effectiveness across diverse settings	Mechanistic interpretability revealing agent assumptions; context-aware adaptation mechanisms	Section 6.3 and Section 7.1

## Data Availability

No new data were created or analyzed in this study.
